# On Compression of the Primitive Carotid Artery in Convulsive Diseases

**Published:** 1841-04-24

**Authors:** 

**Affiliations:** New York


*On Compression of the Primitive Carotid Artery in Convulsive Diseases.* By Dr. Trudeau, of New York.

My object here is not to speak of the compression of the common carotid trunk, or of its ligature in cases of organic derangement such as aneurismal tumours of the cervical region, of the eye, etc., but of the therapeutic use of compression in convulsive diseases, The subject is far from being a new one. but is hardly familiar to medical men,—so much so, that in 1837 Prof. Trousseau published in the Journ. des Connaissares Méd. Chirurg. some cases of arachnitis, in which this treatment was eminently successful. In the meantime, Messrs. Baudelocque and Malapert also came forward with some new facts, claiming the priority. Mr. Dezeimeris then published, to settle the difficulty, a paper in the Expérience, giving a complete history of the subject, and credit where it was deserved. According to him, the first paper on compression of the carotid was written by Parry, of Bath, and published in 1792 by the Medical Society of London. This physician speaks in warm terms of the suggestion, and advises that it be resorted to in those cases in which other remedies have been used without benefit to the patient. Parry acknowledges that he had been led by chance to the employment of this method.

It appears that in a case of accidental lesion of the carotid in which he was compelled to resort to ligature of the vessel, the patient was completely cured of a neuralgic affection of the face, which had baffled the skill of the most eminent physicians of the day. This fact induced him to apply compression in all the neuralgic affections, and it seems with more success than he anticipated.

M. Bland is the first who availed himself of the use of compression of the primitive carotids in meningitis; but he did not pursue his researches on this subject. Liston, who seems to have had no knowledge of Mr. Parry's work, published a case of an obstinate maxillary neuralgia, which was cured by ligature of the carotid. Preston, an English physician of Calcutta, tied the carotid with success in cases of epilepsy. But though M. Trousseau is not the originator, he has paid more attention than any one to this subject, and so far been more successful. Dr. Strochlin, my friend and fellow student, a pupil of Trousseau, and consequently full of the ideas of his master, wrote in 1840 an excellent inaugural dissertation on the employment of compression of the carotids in convulsive affections. So much for the literature of our subject, nothing else having been published since.

These last papers drew the attention of some of the profession to the advantages that might be derived from compression; unfortunately, however, the discovery did not attract much attention, and actually very few physicians know what immense advantage can be derived from it. My object here is not to give a full account of all the cases in which the above named authors have made use of this compression with more or less benefit. Those who are anxious to examine more deeply the subject, will refer to M. Dezeimeris's paper, and to Dr. Strochlin's thesis.

All the cases of convulsive congestion caused by a determination of blood to the brain, without any disorder, as softening, contusion, tumours, etc., are described by MM. Strochlin and Trousseau as immediately relieved by compression. Thus, in arachnitis, epilepsy, and neuralgia, it is deemed the most useful.

In those cases where the compression of the carotids prevents the access of blood to the brain, this organ is *immediately* thrown into an anemic state: and this effect is obtained much more quickly and more safely than by revulsion or depletion. The cerebral circulation is very much diminished, and the want of blood eventually destroys the irritation that had caused the rush towards the encephalon.

In epilepsy, especially, it seems to be particularly beneficial. Parry relates several successful cases. Mr. Earle, according to John Cook, met with similar results: M. Boileau, quoted by Dr. Strochlin, says, that having in a wound of the internal carotid artery, performed the ligature of the vessel on an epileptic patient, he was cured of his dreadful affection. Preston, of Calcutta, relates several cases of the same kind in which he effected a durable cure by the same means. So convinced is Prof. Trousseau of its efficacy that he has always said that in case he or any of his children should be attacked with epilepsy, he would recommend the ligature of the vessel.

M. Strochlin, in his thesis, relates two cases in which compression of both carotids used to stop the epileptic fits almost immediately; and when practised before the attack, succeeded always in preventing it. The following case, which happened a few days ago in my practice, has convinced me of the real efficacy of compression.

Mr. C., 45 Dey-street, æt. fifty-one, a baker. Constitution debilitated by the nature of his trade and the use of ardent spirits; subject since the age of twelve to epileptic fits; three or five attacks during the course of the year; was seized with one of his fits while I was in the house attending another patient. I went immediately to his aid, and found him under a violent attack. Though not a vigorous man, several of his friends could hardly restrain him on his bed. I was told by his family that the attack lasted usually about forty minutes. I have always, when called to assist sufferers by the disease, deeply regretted the insufficiency of medical science, and now determined to give a fair trial to compression. I immediately applied my thumbs on the vessels of both sides, and effected an efficient compression. The face became pale almost immediately; convulsions were less and less violent; the fit was ended. I kept my thumbs strictly applied; the next fit did not occur; the patient recovered his senses and, as in all the cases related by the authors above quoted, complained of the pain caused by the pressure of my fingers. In this case an attack that used to last forty minutes, was stopped in less than ten minutes. Of course, this is but a single fact, and no conclusion can be drawn from it. But this fact, added to all those already published, is, I think, sufficient to direct the attention of the profession towards this powerful agent. Of course, I would never advise to resort to ligature of the carotid in cases of epilepsy. This disease is not yet known enough to justify such violent means, which, in case of failure, might throw some discredit on the physician. But compression can do no harm; it is simple and easily effected. The physician here, instead of folding his arms, or prescribing some vulgar remedy which cannot stop the convulsions, now has in his power to control completely the attack.

Why not employ compression in all the other neroses? In tetanus, to which some cases of epilepsy are so closely allied,—in hydrophobia, if a case of that frightful affection ever occurs to me, I am determined to resort to the ligature of the carotids. In this disease the patient being unavoidably doomed to death, the physician *must* resort to some other means than those proposed in books, which have constantly failed, and remain the opprobrium of science; and analogy here would be strongly in his favour.

In the hysteric fits which occur in pregnant women, and in all the epileptiform convulsions, classified by authors under the denomination of eclampsy, it is also highly serviceable. Dr. S. relates in his thesis cases in which the effect was astonishing. I never had occasion to try it in such cases, and therefore cannot speak from my own experience.

In meningitis and convulsions of children, its efficacy seems well established from Dr. S.'s observations. Several cases which have occurred lately in my practice, have given me a fair chance of testing its efficacy, one of which I will briefly relate.

*Case of Acute Meningitis—Convulsions prevented by Compression—Cure.*

Caroline H., 68 Broome street, aged five; weak and disposed to scrofulous engorgements; born in America, of a German father and a French mother, in circumstances of life rather penurious. Had on the scalp an eruption of impetigo simulating the porrigo favosa. Her parents being anxious to rid her of the disagreeable disease, applied to a certain quack in high renown for the treatment of these diseases. A few days after the use of preparations of lead and frictions with the tar ointment, the eruption disappeared almost suddenly, and the same night the child was seized with chills of great severity, succeeded by a violent fever. Called the following evening. Two hours after I found the little sufferer in a state of torpor; pupils contracted; left eye squinting slightly inwards; does not recognise any one; groans; elevates her hands above her head. I was examining the pulse when the child was seized with violent convulsions. I immediately resorted to compression of both carotids, and kept my fingers applied for half an hour. The eye which squinted was righted in less than two minutes; convulsions diminished gradually; and had I not been compelled by fatigue to remove my fingers, I might have prevented another fit which occurred while we were leeching the child. I compressed again, and in a few minutes stopped the convulsive fit. The leeching was then ended, and blood allowed to flow freely. The following morning I heard that no fit had taken place. The child was in a state of collapse, but the convulsions did not occur again. An abundant pustular eruption, which was determined on the scalp by tartar emetic ointment, arrested the further progress of the disease.

This case seems to be strongly in favour of compression; though the other means which were used in the meantime, as bleeding, revulsives, and cathartics, and especially the restoration of the exanthema, had no doubt a more powerful influence towards the final crisis. Neither do I mean that compression alone ought to be resorted to. But here the effect produced on the convulsive motions is self-evident, and has undoubtedly contributed to the successful termination of the case; and, besides, M. Trousseau's observations are also decidedly in its favour. This is not the only case of convulsions of children in which I have succeeded in arresting the fit. But the above case is, I think, better calculated to illustrate its effects.

It is almost unnecessary to mention that to obtain any favourable result from the use of compression, it is indispensable that it should be well practised. If any compression is made on the jugular vein, quite a different result than the one anticipated will occur. Compression is easily made on the common trunk of the carotid, on each side of the larynx, the artery being superficial, and applied on a solid plane of muscles resting on the cervical vetebræ. Instruments might be constructed so as to be used with great advantage on the fingers, especially in convulsions of children and meningitis, which require a long-continued pressure.

M. T. advises compression of the vessel on the side opposite the convulsions. In general convulsions, compression is to be made on the two vessels.

My object in publishing this paper is merely to induce those of our professional brethren under whose notice it may fall to try compression in all convulsive affections, and to publish their results for the benefit of science. From my own experience, I do not hesitate to say that it ought to take its place among the most powerful means we possess of combating these diseases. The subject is but little known; a vast field is open.

